# Molecular Characterisation of *M. kansasii* Isolates by Whole-Genome Sequencing

**DOI:** 10.3390/pathogens12101249

**Published:** 2023-10-17

**Authors:** Priya Rajendran, Chandrasekaran Padmapriyadarsini, Naveenkumar Nagarajan, Roja Samyuktha, Vadivu Govindaraju, Radhika Golla, Shanmugavel Ashokkumar, Sivakumar Shanmugam

**Affiliations:** ICMR—National Institute for Research in Tuberculosis, Chennai 600031, India; priya.raj@icmr.gov.in (P.R.); padmapriyadarsi.nic@icmr.gov.in (C.P.); vasanthccmb@gmail.com (N.N.); rojasamyuktha312@gmail.com (R.S.); vadivu0219@gmail.com (V.G.); sashokkumarbiotech@gmail.com (S.A.)

**Keywords:** *M. kansasii*, whole-genome sequencing, drug resistance, phylogeny, mutations

## Abstract

*M. kansasii* is the most common non-tuberculous mycobacteria, known to be causing pulmonary and extrapulmonary diseases in humans. Based on molecular methods, *M. kansasii* has been previously classified into seven different subtypes. Now, based on whole-genome sequence analysis, a new species designation was proposed, in which *M. kansasii* species was designated subtype 1 and is of pathogenic significance in both immunocompetent and immunocompromised patients. The aim of the study is to examine the distribution of subtypes, based on whole-genome sequence analysis, and identify the genetic determinants of drug resistance for the isolates. Whole-genome sequencing was performed using 12 isolates for which phenotypic DST results were available. A phylogenetic tree was constructed by alignment of each of the 12 isolates and the additional strains, as well as the *M. kansasii* reference strain, using the MAFFT algorithm. Based on this analysis, all 12 isolates were classified as subtype I. Drug-resistant mutations were identified by analysing the isolates with known drug-resistant loci of MTB and NTM. Although we had mutations in the drug-resistant genes, the significance of those mutations could not be explored due to the minimal availability of data available to compare. Further large-scale studies targeting the phenotypic and genotypic drug-resistance pattern, along with whole-genome analysis, will facilitate a better understanding of the resistance mechanisms involved in *M. kansasii*.

## 1. Introduction

Non-tuberculous mycobacteria (NTM) are environmental organisms and are known to cause opportunistic infection in humans. They comprise more than 180 species and are soon to be revised as more species are added every year [1]. Despite the fact that many of the NTM species are pathogenic, only a few have been demonstrated to have significant associations with human diseases. *M. kansasii* is one such species and is usually graded as the second or third most common NTM isolated from patients with lung diseases [2,3]. In addition to pulmonary diseases, *M. kansasii* is also known to be involved in infection of extrapulmonary sites, including the skin and lymph nodes, and as a disseminated disease. Previously, *M. kansasii* isolates were classified into seven different subtypes based on PCR and restriction fragment length polymorphisms (RFLPs) of the gene hsp65 [4]. Now, based on whole-genome sequence analysis, it has been demonstrated that each subtype, except subtype 7, parallels new species-level lineages of the *M. kansasii* complex [5]. Among the new species designated, the *M. kansasii* species comprises subtype 1 and is of pathogenic significance in both immunocompetent and immunocompromised patients [6], while *M. persicum*, formerly known as subtype 2, is isolated mainly from HIV-infected patients [5,7]. Other four species, namely *M. pseudokansasii* (subtype 3), *M. ostraviense* (subtype 4), *M. innocens* (subtype 5), and *M. attenuatum* (subtype 6) are known to be colonisers with no evidence of causing disease in humans [4,5]. Hence, when *M. kansasii* is identified during NTM diagnosis, insisting further subtype identification will help in understanding its pathogenic significance and appropriate patient management.

According to the ATS/ERS/ESCMID/IDSA guidelines in 2020, treatment of patients with rifampicin (RIF) who are susceptible to *M. kansasii* pulmonary disease includes a regimen of RIF, ethambutol (EMB), and either isoniazid (INH) or macrolide (either clarithromycin (CLR) or azithromycin) (conditional recommendation). On the contrary, patients with RIF-resistant *M. kansasii* or intolerance to one of the first-line drugs, fluoroquinolone (e.g., moxifloxacin) is suggested as part of a second-line regimen. [8]. As per CLSI guidelines, drug-susceptibility testing (DST) for *M. kansasii* is usually carried out for RIF and CLR. RIF resistance (minimum inhibitory concentration (MIC) > 2 µg/mL) is uncommon but can be observed in isolates from patients with prolonged exposure to rifamycin and unsuccessful treatment of a regimen containing rifamycin [9]. Resistance to CLR is determined by an MIC of 32 µg/mL or higher [9]. Susceptibility testing to other drugs (amikacin, ciprofloxacin, doxycycline, linezolid, minocycline, moxifloxacin, rifabutin, and trimethoprim-sulfamethoxazole) is conducted only when RIF resistance is identified [10]. In a study conducted by us previously, we discovered that out of the 18 strains of *M. kansasii* subjected to DST using the Sensititre method (broth microdilution), doxycycline showed the highest resistance pattern with 13 strains, followed by RIF and trimethoprim/sulfamethoxazole with 7 strains. Out of the 22 strains tested using the proportion sensitivity testing (PST) method, 20 and 10 demonstrated resistance to INH and EMB, respectively. However, there was a poor correlation between the resistance pattern of the drugs tested and the clinical outcome of the patients from whom these strains were isolated [11]. In this study, our objective was to undertake whole-genome sequence analysis of these isolates in order to examine potential genetic determinants of drug resistance and study the distribution of subtypes through phylogenetic analysis.

## 2. Materials and Methods

### 2.1. Preliminary Genotypic Identification Tests

**Figure 1:**
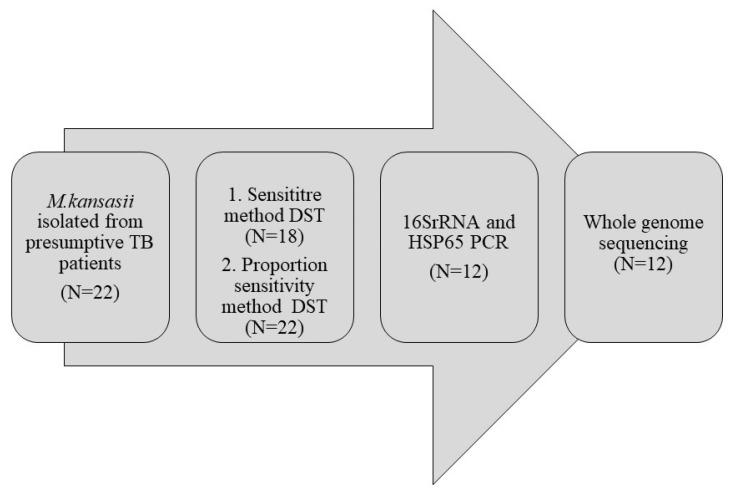
Workflow of experiments carried out for *M. kansasii* isolates.

A total of 12 *M. kansasii* isolates out of 22 reported earlier were available for this study and were subcultured onto LJ medium for further characterisation. Following conventional species identification methods and biochemical tests, molecular tests targeting the *hsp65* and *16SrRNA* gene was carried out. Briefly, the genomic DNA was extracted from LJ medium colonies using the CTAB (cetyltrimethylammonium bromide) extraction method [12]. The extracted DNA was subjected to *16SrRNA* PCR using the primer sequences 5′-ATGCACCACCTGCACACAGG and 5′-GGTGGTTTGTCGCGTTGTTC and *hsp65* PCR using the primer sequences Tbll (5′-ACCAACGATGGTGTGTCCAT) and Tb12 (5′-CTTGTCGAACCG CATACCCT). The reaction mixture and cycling conditions were adapted from the protocol published elsewhere [13,14]. The isolates confirmed by the molecular method were included for whole-genome sequence analysis (Figure 1).

### 2.2. Genome Sequencing

Genomic DNA extracted from clinical isolates using the CTAB was further purified using the Genomic DNA Clean and Concentrator kit (Zymo Research, Irvine, CA, USA). Purified DNA was assessed for quality and quantity using the Nano Drop^TM^ and Qubit^TM^ dsDNA assay kit method (ThermoFisher Scientific, Waltham, MA, USA). Sequencing libraries were prepared using the NEBNext Ultra DNA Library preparation kit. In brief, fragmented DNA was subjected to a series of enzymatic steps to repair the ends and tailing with dA-tail, followed by ligation of adapter sequences. Adapter ligated fragments were cleaned up using SPRI beads, and the clean fragments were indexed using limited cycle PCR to generate final libraries for paired-end sequencing on a HiSeq X 10 sequencer (Illumina, San Diego, Ca, USA). Paired-end libraries were prepared from 1 ng of high-quality genomic DNA with the Nextera XT DNA sample preparation kit according to the manufacturer’s instructions (Illumina Inc., San Diego, USA). The libraries were sequenced on a HiSeq 2500 or a NextSeq 500 instrument (Illumina, San Diego, CA, USA) at a read length of 2 × 150 bp.

### 2.3. Dataset

The dataset of the 12 isolates containing the phenotypic DST results and quality of sequence and alignment is shown in Table 1. The reference sequence of *M. kansasii* subtype was obtained from NC_022663.1. All 12 isolates were mapped to the reference sequence from the start and endpoint of 1 to 6,432,277.

### 2.4. Variant Calling

Open-source programmes, namely Genome Analysis Tool Kit (GATK) [15], BWA [16], SAMtools [17] and PICRD-tools, were employed for variant calling. Raw FASTQ sequences were mapped to the reference genome (*Mycobacterium kansasii* ATCC 12478) using the BWA-MEM algorithm and SAMtools. The mapped reads were sorted, duplicated, and saved in BAM format. GATK was used for the realignment of reads around insertions or deletions. SAMtools mpileup utility was used to call variants with four reads mapped in the forward and reverse direction at an allele frequency of 75% and with a minimum of four calls with a phred score of at least 20.

### 2.5. Phylogenetic Analysis

Consensus genomes for the 12 isolates were generated by a computational tool (iVar) [18] using default settings. Full-length sequences of *16SrRNA*, along with genes such as *rpoB*, *hsp65*, *tuf* and the intergenic spacer region (ITS) of the *M. Kansasii* reference strain, were downloaded from NCBI and were used as a reference for alignments with the respective sequences of 12 isolates (Table 1) and the additional strains reported in other studies (Table 2). Sequences from the same loci of the consensus genomes were identified using the blastn algorithm. The gene sequences of the reference strains, 12 study strains, and the additional strains reported from other studies were concatenated and aligned using the MAFFT algorithm [19]. These alignments were directly given as the input for computing a maximum-likelihood phylogenetic tree using RAxML-NG version 1.1 [20]. RaxML uses the GTR-GAMMA model of nucleotide substitution, bootstrap, and a randomseed value to construct a phylogenetic tree. A best scoring tree with maximum likelihood is identified with bootstrap search. The branch support was provided by 1000 bootstrap replicates. The revised subtype classification data were obtained from previous studies and compared with our sequences for subtyping [4,21].

### 2.6. Identification of Mutations in Drug-Resistant Locus

Since a mutation catalogue exclusively for NTM was not available, intragenic drug-resistant locus of MTB from the WHO mutation catalogue [22,23] and drug-resistant locus available for NTM from the literature were obtained [24]. These known loci were analysed in the 12 consensus genomes to identify drug-resistant mutations. Drug-resistant gene sequences from the consensus genomes were translated using an online server [25], and the non-synonymous mutations were identified by alignment with the *M. Kansasii* reference sequence and the details of the same are provided in the Supplementary Materials.

## 3. Results

### 3.1. Identification by Molecular Methods

**Figure 2:**
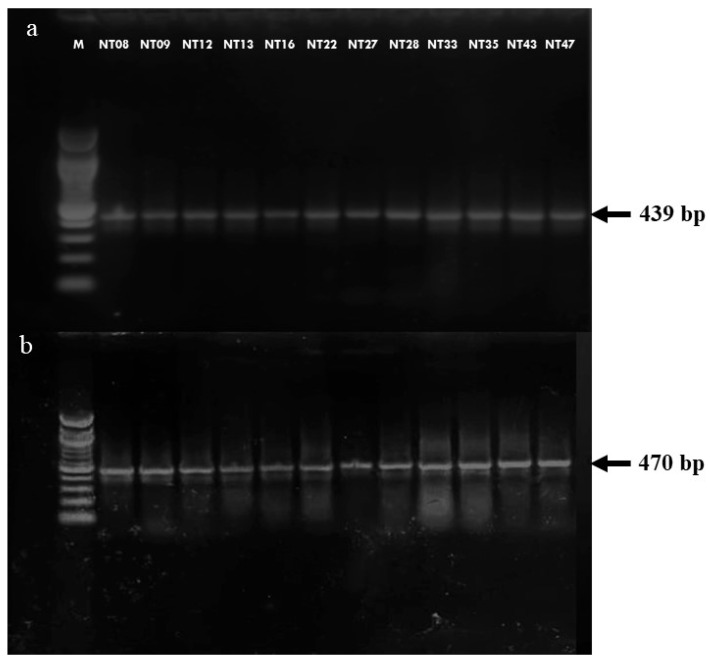
HSP65 PCR (**a**) and 16srRNA PCR (**b**) for the 12 isolates that were subjected to sequencing. Lane M: Marker.

The product of the *16SrRNA* and *hsp65* gene with the size of 470 bp and 439 bp was amplified from all the DNA of culture isolates. The hsp65 PCR products were further subjected to RFLP for species identification of *M. kansasii* (Figure 2).

### 3.2. Subtype Classification

**Figure 3:**
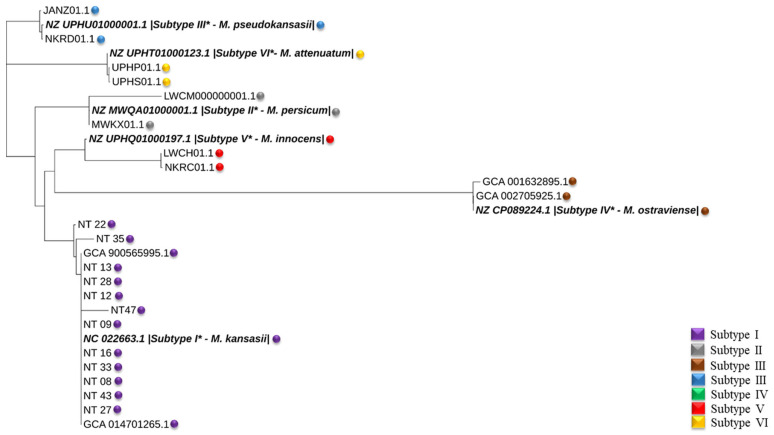
Phylogenetic tree based on 16srRNA, *rpoB*, hsp65, ITS, and *tuf* gene sequences constructed using neighbour-joining method (* former classification).

The phylogenetic tree was constructed using concatenated *16srRNA*, *rpoB*, hsp65, ITS, and *tuf* genes. Based on this analysis, all 12 isolates were classified as subtype I when compared to the reference sequence and sequences obtained from previous studies based on this analysis (Figure 3, Table 2).

### 3.3. Association of Mutation with Drug Resistance

The drug-resistant locus of MTB and *M. kansasii* were aligned with same locus of 12 isolates and some mutations were identified as follows (Table 3).

#### 3.3.1. RIF and INH Resistance

The genes *rpoB* and *katG* associated with RIF and INH resistance, respectively, in MTB were analysed for mutations in the *M. kansasii* isolates. There were no mutations found in comparison with both MTB and *M. kansasii* reference genomes.

#### 3.3.2. EMB Resistance

Sequence analysis of the EMB-resistance-associated loci (*embB* and *embCA*) revealed mutations S272N, S565G, Q853R, and A1007T in the *embB* gene of 10 isolates when compared to the MTB genome. Out of these isolates, five of them were resistant to EMB, identified previously using the PST method of DST. When the sequences were compared to the *M. kansasii* reference sequence, one isolate had 5 non-synonymous mutations in the emb gene (Table 3), and this isolate was resistant to EMB according to the phenotypic method. However, the association of these mutations with drug resistance could not be explored since these mutations were not reported elsewhere.

When another resistance-determining region of EMB, *aftB*, was compared with the MTB reference sequence, three isolates had 9 mutations. Out of these three isolates, one was EMB resistant according to the phenotypic DST. When compared with the *M. kansasii* reference sequence, one isolate had 18 mutations, and this isolate was sensitive according to the phenotypic DST (Table 3).

#### 3.3.3. Clarithromycin Resistance

Three isolates that were resistant to CLR according to the phenotypic DST did not have any significant mutations when compared with *M. kansasii* and the MTB reference genome. However, an isolate that was susceptible to CLR had a mutation in the *rrl* gene at a resistance-determining region (A2089G) when compared to the *M. kansasii* reference genome.

#### 3.3.4. Quinolone Resistance

Two isolates that were previously reported to be resistant to ciprofloxacin and one isolate to moxifloxacin according to phenotypic DST had no mutations in the quinolone resistance-determining region (QRDR) of the *gyrA* and *gyrB* loci when compared with both MTB and the *M. kansasii* reference genome.

#### 3.3.5. Amikacin Resistance

The sequences of two isolates that were resistant to Amikacin according to phenotypic DST when compared with the MTB sequences at *eis* genes, no mutations were found. However, there were three non-synonymous mutations (V301I, E348D, D352G) in two other isolates that were sensitive according to phenotypic DST. When compared with the *M. kansasii* reference sequence, two non-synonymous mutations were found (M293T, V297I) for these isolates.

## 4. Discussion

Of the NTM diseases reported worldwide, next to the *M. avium* complex and *M. abscessus*, *M. kansasii* is known to be the predominant organism isolated from pulmonary infections in a different geographical area [26,27]. While the prevalence of NTM in India ranged from 0.7% to 34% [28], distribution of *M. kansasii* among them ranged from 1.5% to 11.8% [29]. Although distribution of *M. kansasii* isolates have been documented throughout the country, further subtype analysis was not carried out in many studies. In our earlier studies, we reported the isolation of *M. kansasii* (46.8%) from presumptive pulmonary TB patients and its drug-resistance pattern via phenotypic DST. In this study, we carried out molecular analysis of these isolates [11,30] by hsp65 PCR and 16srRNA PCR and further phylogenetic analysis of the whole-genome sequences.

Out of the 18 *M. kansasii* isolates obtained, 12 were subjected to whole-genome sequencing, and all the isolates were grouped as subtype I (*M. kansasii* as per recent classification) [4]. The finding concurs with the fact that *M. kansasii* is associated with human disease in both immunocompetent and immunocompromised hosts, and in our study, all the patients were immunocompetent. Studies from other parts of the world like China, Europe, the United States, and Japan have also documented the role of subtype I in human infections [21,31], irrespective of the site of infection. Another study from China documented minimal distribution of subtypes (II, III and IV), along with the majority of subtype I associated with pulmonary disease [32].

As per CLSI guidelines, drug-susceptibility testing (DST) for *M. kansasii* is usually carried out for RIF and CLR. We reported a total of seven RIF-resistant isolates earlier by phenotypic DST in which the MIC was 2 µg/mL for three and 8 µg/mL for four isolates. In the *rpoB* gene of these isolates when compared to MTB and the *M. kansasii* reference genome, no mutations were found. Klein et al., have reported that mutations in the rifampin-resistant isolates (n = 5) appeared to be associated with high-level resistance (256 mg/mL). This could be a reason for the RIF-resistant isolates not having any mutations conferred in the *rpoB* gene. Moreover, other factors like role of the efflux pump and the persistent nature of bacilli due to prolonged exposure to the drug also need to be explored. Further genotypic studies are also needed to confirm the correlation between the mutations and high level of RIF resistance to demonstrate its clinical significance.

We could not find any mutations associated with INH resistance for all the isolates (n = 12) that were resistant to this drug by phenotypic DST. While the INH resistance in MTB is attributed to the role of mutations in the *inhA* and *katG* gene, studies documenting the role of these genes leading to INH resistance among NTM is absent [24,33].

Since *embB* and *embCA* genes are associated with EMB resistance in MTB, we looked for mutations in these genes with comparison to MTB and the *M. kansasii* reference genome for all 12 isolates. A total of ten isolates (five resistant and five susceptible by phenotypic DST) revealed four mutations when compared with the MTB genome, and one isolate (EMB resistant by phenotypic DST) had five mutations in comparison to the *M. kansasii* reference genome. This interesting concurrence between phenotypic and genotypic results for some isolates implicates the need for further genetic studies exploring the association of these mutations with drug resistance. Another study by Bakula et al., reported M306I, G406P, and M423I amino acid substitutions, which were associated with EMB resistance in MTB but not yet reported in NTM [24]. Moreover, as these mutations were found in both EMB-resistant and EMB-susceptible isolates, their specificity for EMB resistance in *M. kansasii* was not warranted.

*M. kansasii* isolates that were found to be resistant to CLR previously by phenotypic DST did not have mutations in the *rrl* gene. In contrast, an isolate susceptible to CLR was found to have a single mutation (A2089G) in the drug-resistant locus. Such a type of single but different mutation (A2266C) in the *rrl* gene has been reported in a study by Bakula et al., but concurring with phenotypic resistance [24]. However, a study from China reported a mix of concordance patterns where 9 CLR-resistant isolates had mutations at position 2058 and 2059 and discordance patterns where 16 CLR-resistant isolates were not found to have mutations in the *rrl* gene [32].

The mechanism of drug resistance in NTM could be either acquired in that it is mediated by drugs or by being inherently mediated by the efflux pump or porin channels present in the cell envelope. These porin channels present in the cell envelope may help in the low permeability of the cell wall to external stress due to drugs, resulting in a resistant phenotype [34]. Such mechanisms involved in NTM drug resistance need to be explored through whole-genome sequence analysis studies with a larger set of samples.

A major limitation of this study is the low number of isolates that were subjected to sequencing for the determination of drug-resistance genes. Another limitation is although we could identify few mutations associated with drug resistance, the phenotypic or genotypic drug-resistance patterns could not be correlated with treatment outcome. Without this correlation, it is difficult to mention if the mutation seen in sequence analysis has any relevance.

## 5. Conclusions

To our knowledge, this study is the first report from India on whole-genome sequence analysis of *M. kansasii* on its subtype distribution and drug-resistance association. Although we had many mutations in the drug-resistant genes, with the given minimal number of such studies published elsewhere, we could not compare these mutations (except for few) to explore its significance. Further studies involving both drug-susceptibility testing and whole-genome sequence analysis of *M. kansasii* isolates inclusive of structural inferences are needed to understand the molecular mechanisms involved in drug resistance and to further demonstrate its clinical significance.

## Figures and Tables

**Table 1:** Mapping and coverage of NTM isolates with *M. kansasii* reference sequence.

S. No.	Lab No.	Resistance to Drugs by Phenotypic DST	Gene Mutations Comparison		Treatment Outcome	Alignment Coverage		
			MTB Genome	*M. kansasii* Genome		No. of Reads	Coverage	Mean Depth
1	NT 08	RIF/SXT/DOX	*embB*	No mutation	Died	5,067,055	99.6502	38.1
2	NT 09	EMB	*embB*	No mutation	Cured	4,350,931	99.7226	38.2
3	NT 12	CLR/DOX	*embB*, *aftB*	*aftB*	Cured	4,199,386	98.8226	37.9
4	NT 13	RIF/SXT/DOX/AMK/LZD/CIP/EMB	*embB*, *aftB*	No mutation	Relapse	4,701,856	98.8198	38.1
5	NT 16	No resistance	*embB*, *eis*	*eis*	Cured	4,740,116	99.6771	37.9
6	NT 22	EMB	*embB*	*embB*	Cured	4,626,353	99.3018	38
7	NT 27	DOX/EMB	*embB*	No mutation	Died	4,862,099	99.9468	38
8	NT 28	SXT/DOX/ EMB	*embB*, *eis*	No mutation	Cured	4,557,193	99.6895	38.1
9	NT 35	No resistance	*aftB*	No mutation	Cured	3,844,920	99.3199	38.3
10	NT 43	CLR/RFB/RIF/SXT/AMK/ DOX	*embB*	No mutation	Cured	4,094,943	99.6353	38.1
11	NT 33	No resistance	*embB*	No mutation	Cured	4,372,578	99.6714	38.1
12	NT 47	EMB	No mutation	*rrl*	Cured	1,856,345	99.5578	40.9

**Table 2:** Accession details of *M. kansasii* genomes used in phylogenetic analysis (* former classification).

S. No.	Strain	Former *kansasii* Subtype	GenBank No.
1	ATCC 12478	(*M. kansasii*) I	NC_022663.1_I *
2	MK7	(*M. kansasii*) I	GCA_900565995.1
3	Kuro-I	(*M. kansasii*) I	GCA_014701265.1
4	12MK	(*M. persicum*) II	NZ_MWQA01000001.1_II *
5	1010001469	(*M. persicum*) II	LWCM00000000.1
6	3MK	(*M. persicum*) II	MWKX01.1
7	MK142	(*M. pseudokansasii*) III	NZ_UPHU01000001.1_III *
8	732	(*M. pseudokansasii*) III	JANZ01.1
9	174_15_11	(*M. pseudokansasii*) III	NKRD01.152
10	FDAARGOS_1613	(*M. ostraviense*) IV	NZ_CP089224.1_IV *
11	241/15	(*M. ostraviense*) IV	GCA_002705925.1
12	1010001458	(*M. ostraviense*) IV	GCA_001632895.1
13	MK21	(*M. innocens*) V	NZ_UPHQ01000197.1_V *
14	49_11	(*M. innocens*) V	NKRC01.1
15	1010001454	(*M. innocens*) V	LWCH01.1
16	MK41	(*M. attenuatum*) VI	NZ_UPHT01000123.1_VI *
17	MK191	(*M. attenuatum*) VI	UPHS01.1
18	MK136	(*M. attenuatum*) VI	UPHP01.1

**Table 3:** List of mutations identified in the study isolates in correspondence to MTB and *M. kansasii* reference genome.

Target Drug	Drug-Resistant Locus	Mutation in Comparison to MTB Reference Sequence	Mutation in Comparison to *M. kansasii* Reference Sequence
Amikacin	*eis*/MKAN_RS04925	V301I	M293T
		E348D	V297I
		D352G	
Ethambutol	*embB*	S272N	L78M
		S565G	G130A
		Q853R	A159G
		A1007T	A259T
			Y737N
	*aftB*	S159A	V100A
		I202V	V107A
		S238G	M127V
		R401H	A133V
		M491L	M192L
		V511A	F257L
		A516Q	M331V
		A524G	V339L
		Q561R	I354V
			K399R
			V393L
			L394S
			G412V
			E435D
			T515S
			I541L
			K603R
			S657P

## Data Availability

Not applicable.

## Supplementary Materials

The following supporting information can be downloaded at: https://www.mdpi.com/article/10.3390/pathogens12101249/s1, Supplementary file: Alignment of isolates with *M. kansasii* reference sequence showing mutation in drug resistant loci.
