# Supplementary material for: Molecular Characterisation of M. kansasii Isolates by Whole-Genome Sequencing
Source: Pathogens. 2023 Oct 17;12(10):1249. doi: 10.3390/pathogens12101249 (PMC10610326; doi:10.3390/pathogens12101249)
Supplement: Supplementary file 1 [file pathogens-12-01249-s001.zip › pathogens-2554915-supplementary.pdf]

**Supplementary file: Alignment of isolates with *M. kansasii* reference sequence showing mutation in drug resistant loci.**

**Alignment of *eis* gene between *M. kansasii* and NT16.**

```
NT16               1 MYFLAAASFPDFMGPRPANTWRSLVPADGAVVSCDGGDVVGMALYLDLRL      50
                     ||||||||||||||||||||||||||||||||||||||||||||||||||
M.kansasii         1 MYFLAAASFPDFMGPRPANTWRSLVPADGAVVSCDGGDVVGMALYLDLRL      50

NT16              51 TVPGGAVLPAAGLSFVAVAPTHRRRGLLRLMCAELHRRISDSEYPMAALY     100
                     ||||||||||||||||||||||||||||||||||||||||||||||||||
M.kansasii        51 TVPGGAVLPAAGLSFVAVAPTHRRRGLLRLMCAELHRRISDSEYPMAALY     100

NT16             101 ASEGGIYGRFGYGPATVLQELTLDRRFARFHLDAPDGGRVRLVNPAEHRD     150
                     ||||||||||||||||||||||||||||||||||||||||||||||||||
M.kansasii       101 ASEGGIYGRFGYGPATVLQELTLDRRFARFHLDAPDGGRVRLVNPAEHRD     150

NT16             151 EFAAIYERWRQQVPGGLARPDVLWEDALGECTNSPRGDTRLFAFLHTDGY     200
                     ||||||||||||||||||||||||||||||||||||||||||||||||||
M.kansasii       151 EFAAIYERWRQQVPGGLARPDVLWEDALGECTNSPRGDTRLFAFLHTDGY     200

NT16             201 ALYRVDATDQKLARVEELRAVTADAHISLWRALLGLDSMERVRVITHPDD     250
                     ||||||||||||||||||||||||||||||||||||||||||||||||||
M.kansasii       201 ALYRVDATDQKLARVEELRAVTADAHISLWRALLGLDSMERVRVITHPDD     250

NT16             251 PLPFLLTDTRLASPTFRQDGLWLRIMDIPAALEARGYCADLATVIGISDG     300
                     ||||||||||||||||||||||||||||||||||||||||||.|||:|||
M.kansasii       251 PLPFLLTDTRLASPTFRQDGLWLRIMDIPAALEARGYCADLAMVIGVSDG     300

NT16             301 GRYRLQIRDGRALCSPTDASADVVLGRDVLGSLYLGVHRASTLAAANRLR     350
                     ||||||||||||||||||||||||||||||||||||||||||||||||||
M.kansasii       301 GRYRLQIRDGRALCSPTDASADVVLGRDVLGSLYLGVHRASTLAAANRLR     350

NT16             351 TRDSGLVNRLDAAFASDVPAQTAFEF    376
                     ||||||||||||||||||||||||||
M.kansasii       351 TRDSGLVNRLDAAFASDVPAQTAFEF    376
```

**Alignment of *embB* gene between *M. kansasii* and NT22.**

```
NT22               1 MSMTTASRDVRVTRWVATIAGLVGFVLSVATPLLPVVQTTAMLNWPQNGQ      50
                     ||||||||||||||||||||||||||||||||||||||||||||||||||
M.kansasii         1 MSMTTASRDVRVTRWVATIAGLVGFVLSVATPLLPVVQTTAMLNWPQNGQ      50

NT22              51 LNSVTAPLITLTPVDLTATVPCEVVRGMPPQGGVVLGTAPKQGKDANLQA     100
                     |||||||||||||||||||||||||||:||||||||||||||||||||||
M.kansasii        51 LNSVTAPLITLTPVDLTATVPCEVVRGLPPQGGVVLGTAPKQGKDANLQA     100

NT22             101 MFVVVSSQRVDVTDRNVVILSVPRDQVVGAANAPGCSSIEVTSTHAGTFA     150
                     |||||||||||||||||||||||||||||.||||||||||||||||||||
M.kansasii       101 MFVVVSSQRVDVTDRNVVILSVPRDQVVGGANAPGCSSIEVTSTHAGTFA     150

NT22             151 TFVGLKDPGGQPLRGGFPDPNLRPQIVGVFTDLTGPAPPGLKLSATIDTR     200
                     ||||||||.|||||||||||||||||||||||||||||||||||||||||
M.kansasii       151 TFVGLKDPAGQPLRGGFPDPNLRPQIVGVFTDLTGPAPPGLKLSATIDTR     200

NT22             201 FSTTPTTLKLLAMVGAIVATVVALVALWRLDRLDGHRMRRWIPQNWRTFT     250
```

```
                       ||||||||||||||||||||||||||||||||||||||||||||||||||
M.kansasii         201 FSTTPTTLKLLAMVGAIVATVVALVALWRLDRLDGHRMRRWIPQNWRTFT    250

NT22               251 LLDGVVIFTFLLWHVIGANSSDDGYILGMARVADRAGYMSNYFRWFGSPE    300
                       ||||||||.|||||||||||||||||||||||||||||||||||||||||
M.kansasii         251 LLDGVVIFAFLLWHVIGANSSDDGYILGMARVADRAGYMSNYFRWFGSPE    300

NT22               301 DPFGWYYNLLALMTHVSDASIWMRLPDLFAGLVCWLLLSREVLPRLGPAV    350
                       ||||||||||||||||||||||||||||||||||||||||||||||||||
M.kansasii         301 DPFGWYYNLLALMTHVSDASIWMRLPDLFAGLVCWLLLSREVLPRLGPAV    350

NT22               351 TSSKAANWAAATVLLTAWMPFNNGLRPEGIIALGSLVTYVLIERSMRYSR    400
                       ||||||||||||||||||||||||||||||||||||||||||||||||||
M.kansasii         351 TSSKAANWAAATVLLTAWMPFNNGLRPEGIIALGSLVTYVLIERSMRYSR    400

NT22               401 LTPAALAIIAAAFTLGVQPTGLIAVAALVAGGRPILRIFVKRHRLVGTLP    450
                       ||||||||||||||||||||||||||||||||||||||||||||||||||
M.kansasii         401 LTPAALAIIAAAFTLGVQPTGLIAVAALVAGGRPILRIFVKRHRLVGTLP    450

NT22               451 LLSPMLAAGTIILTVVFADQTLSTVLEATRVRGKIGPSQAWYTENLRYYY    500
                       ||||||||||||||||||||||||||||||||||||||||||||||||||
M.kansasii         451 LLSPMLAAGTIILTVVFADQTLSTVLEATRVRGKIGPSQAWYTENLRYYY    500

NT22               501 LILPTVDGSLSRRFGFLITALCLFTAVFIMLRRKRIPGVARGPAWRLMGV    550
                       ||||||||||||||||||||||||||||||||||||||||||||||||||
M.kansasii         501 LILPTVDGSLSRRFGFLITALCLFTAVFIMLRRKRIPGVARGPAWRLMGV    550

NT22               551 IFATMFFLMFTPTKWVHHFGLFAAVGAAMAALTTVLVSPSVLRWSRNRMA    600
                       ||||||||||||||||||||||||||||||||||||||||||||||||||
M.kansasii         551 IFATMFFLMFTPTKWVHHFGLFAAVGAAMAALTTVLVSPSVLRWSRNRMA    600

NT22               601 FLAAVLFMLALCWATTNGWWYVSSYGVPFNSAMPKIAGITVSTIFFALFA    650
                       ||||||||||||||||||||||||||||||||||||||||||||||||||
M.kansasii         601 FLAAVLFMLALCWATTNGWWYVSSYGVPFNSAMPKIAGITVSTIFFALFA    650

NT22               651 IAAVYAAWLHFAPRGSGEGRLTRALTWPSQAPVPLAAGFMAVVFVASMVA    700
                       ||||||||||||||||||||||||||||||||||||||||||||||||||
M.kansasii         651 IAAVYAAWLHFAPRGSGEGRLTRALTWPSQAPVPLAAGFMAVVFVASMVA    700

NT22               701 GIVRQYPTYSNGWSNLRAFVGGCGLADDVLVEPDPNNGFMTALPGDYGPL    750
                       ||||||||||||||||||||||||||||||||||||.|||||||||||||
M.kansasii         701 GIVRQYPTYSNGWSNLRAFVGGCGLADDVLVEPDPNYGFMTALPGDYGPL    750

NT22               751 GPLGGTNPTGFTPNGVPEHTVAEAIVMKPNQPGTDYDWDAPTKLKTAGIN    800
                       ||||||||||||||||||||||||||||||||||||||||||||||||||
M.kansasii         751 GPLGGTNPTGFTPNGVPEHTVAEAIVMKPNQPGTDYDWDAPTKLKTAGIN    800

NT22               801 GSTVPLPYQLDPARVPLAGTYTTGAQRQSKLASAWYLLPTPDDGHPLVAV    850
                       ||||||||||||||||||||||||||||||||||||||||||||||||||
M.kansasii         801 GSTVPLPYQLDPARVPLAGTYTTGAQRQSKLASAWYLLPTPDDGHPLVAV    850

NT22               851 TAAGKIAGHSVLHGYTPGQTVVLEYARPGPGALVPAGRLVPDDLYGEQPK    900
                       ||||||||||||||||||||||||||||||||||||||||||||||||||
M.kansasii         851 TAAGKIAGHSVLHGYTPGQTVVLEYARPGPGALVPAGRLVPDDLYGEQPK    900

NT22               901 AWRNLRFARDKMPADAVAVRVVAEDLSLTPEDWIAVTPPRVPDLRSLQEY    950
                       ||||||||||||||||||||||||||||||||||||||||||||||||||
M.kansasii         901 AWRNLRFARDKMPADAVAVRVVAEDLSLTPEDWIAVTPPRVPDLRSLQEY    950

NT22               951 VGSTQPVLLDWAVGLAFPCQQPMLHVNGVTEIPKFRITPDYNAKKLDTDT   1000
                       ||||||||||||||||||||||||||||||||||||||||||||||||||
M.kansasii         951 VGSTQPVLLDWAVGLAFPCQQPMLHVNGVTEIPKFRITPDYNAKKLDTDT   1000

NT22              1001 WEDGVNGGLLGITDLLLRAHVMATYLSRDWARDWGSLRKFDTLVDAPPAQ   1050
```

```
                      ||||||||||||||||||||||||||||||||||||||||||||||||||
M.kansasii      1001 WEDGVNGGLLGITDLLLRAHVMATYLSRDWARDWGSLRKFDTLVDAPPAQ   1050

NT22            1051 LDLGTATRSGLWSPGKIRIGP   1071
                     |||||||||||||||||||||
M.kansasii      1051 LDLGTATRSGLWSPGKIRIGP   1071
```

**Alignment of *aftB* gene between *M. kansasii* and NT12.**

```
M.kansasii         1 MGPLLPSASSRFKALRASALARRPAVRRARWPVFPYEPVVRISLWVSVAV     50


NT12               1 --------------------------------------------------      0

M.kansasii        51 VCMLFGWGAWQRRWIADDGLIVLRTVRNLLAGNGPVFNMGERVEANTSTV    100
                                                           |||||||||||.
NT12               1 --------------------------------------MGERVEANTSTA     12

M.kansasii       101 WTYLLYVASWVGGPMRLEYVALAVALMLSLLGAALLMLGTGRLYAPSLRG    150
                     ||||||.||||||||||||||||||||:|||||.||||||||||||||||
NT12              13 WTYLLYAASWVGGPMRLEYVALAVALVLSLLGVALLMLGTGRLYAPSLRG     62

M.kansasii       151 RRAIMLPAGALVYIAVPPARDFATSGLESGLVLTYLGLLWWMMVCWAQPL    200
                     |||||||||||||||||||||||||||||||||||||||||:||||||||
NT12              63 RRAIMLPAGALVYIAVPPARDFATSGLESGLVLTYLGLLWWLMVCWAQPL    112

M.kansasii       201 RVRPHGRVFIGALAFVAGCSVLVRPELALMGGLALIMMLVAARTWRRRVL    250
                     ||||||||||||||||||||||||||||||||||||||||||||||||||
NT12             113 RVRPHGRVFIGALAFVAGCSVLVRPELALMGGLALIMMLVAARTWRRRVL    162

M.kansasii       251 IVVAGGFLPVAYQIFRMGYYALLVPGTALAKDAAGDKWSQGMIYLSNFNR    300
                     ||||||.|||||||||||||||||||||||||||||||||||||||||||
NT12             163 IVVAGGLLPVAYQIFRMGYYALLVPGTALAKDAAGDKWSQGMIYLSNFNR    212

M.kansasii       301 PYALWVPIVLLVPLGLVLMLARRRPSFLRPMVAPDYGRVARAVQSPAAVV    350
                     ||||||||||||||||||||||||||||||:|||||||:|||||||||||
NT12             213 PYALWVPIVLLVPLGLVLMLARRRPSFLRPVVAPDYGRLARAVQSPAAVV    262

M.kansasii       351 AFMIGSGLLQALYWIRQGGDFMHGRVLLAPLFCLLAPVAVIPVLLPDGKD    400
                     |||:||||||||||||||||||||||||||||||||||||||:.||||:|
NT12             263 AFMVGSGLLQALYWIRQGGDFMHGRVLLAPLFCLLAPVAVIPLSLPDGRD    312

M.kansasii       401 FSKETGYWLAGGVSILWLGVAGWSLWAANSPGMGDDATHVTYTGIVDERR    450
                     |||||||||||.||||||||||||||||||||||||||||||||||||||
NT12             313 FSKETGYWLAGVVSILWLGVAGWSLWAANSPGMGDDATHVTYTGIVDERR    362

M.kansasii       451 FYAQATGHAHPLTAADYLDYPRMAAVLTALDNTPEGALLLPSGNYNQWDL    500
                     ||||||||||||||||||||||||||||||||||:|||||||||||||||
NT12             363 FYAQATGHAHPLTAADYLDYPRMAAVLTALDNTPDGALLLPSGNYNQWDL    412

M.kansasii       501 VPMIPPGTAPGIPATQKPQHAVFFTNLGMLGMNVGLDVRVIDQIGLANPL    550
                     ||||||||||||||:||||||||||||||||||||||||||:||||||||
NT12             413 VPMIPPGTAPGIPASQKPQHAVFFTNLGMLGMNVGLDVRVLDQIGLANPL    462

M.kansasii       551 AQHTERLKHGRIGHDKNLFPDWVIADGPWVKWYPGVPGYLDPAWVAQAEA    600
                     ||||||||||||||||||||||||||||||||||||||||||||||||||
NT12             463 AQHTERLKHGRIGHDKNLFPDWVIADGPWVKWYPGVPGYLDPAWVAQAEA    512

M.kansasii       601 ALKCPATQAVLNSVVRAPLTLRRFVSNVVHSFEFTRYRIDRVPLNELIRCG    650
                     ||:||||||||||||||||:||||||||||||||||||||||||||||||
```

```
NT12              513 ALRCPATQAVLNSVRAPLTVRRFVSNVVHSFEFTRYRIDRVPLNELIRCG    562

M.kansasii        651 LEVPDVSPAPARE    663
                      ||||||.||||||
NT12              563 LEVPDVPPAPARE    575
```

**Alignment of *rrl* loci between *M. kansasii* and NT47.**

Drug resistant location with A2089G mutation found in one of our isolate NT47 results highlighted in blue rectangle, mutation is marked in red color.

```
M.kansasii          1 TTGTAAGTGTCTAAGGGCGCATGGTGGATGCCTTGGCATCGAGAGCCGAT     50
                      |||||||||||||||||||||.||||||||||||||||||||||||||||
NT47                1 TTGTAAGTGTCTAAGGGCGCACGGTGGATGCCTTGGCATCGAGAGCCGAT     50

M.kansasii         51 GAAGGACGTGGGAGGCTGCGATAAGCCTCGGGGAGCTGTCAACCGAGCGT    100
                      ||||||||||||||||||||||||||||||||||||||||||||||||||
NT47               51 GAAGGACGTGGGAGGCTGCGATAAGCCTCGGGGAGCTGTCAACCGAGCGT    100

M.kansasii        101 GGATCCGAGGATTTCCGAATGGGGAAACCCAGCACGAGTGATGTCGTGTT    150
                      |||||||||||||||||||||||||||||||.|||||||.||||||||||
NT47              101 GGATCCGAGGATTTCCGAATGGGGAAACCCAACACGAGTTATGTCGTGTT    150

M.kansasii        151 ACCCGCATCTGAATATATAGGGTGCGGGAGGGAACGCGGGGAAGTGAAAC    200
                      |||||||||||||||||||||||||||||||||||||||||||.||||||
NT47              151 ACCCGCATCTGAATATATAGGGTGCGGGAGGGAACGCGGGGAACTGAAAC    200

M.kansasii        201 ATCTCAGTACCCGTAGGAGAAGAAAACAAAAGTGATTCCGTAAGTAGTGG    250
                      ||||.|||||||||||||||||||||||||||||||||||||||||||||
NT47              201 ATCTTAGTACCCGTAGGAGAAGAAAACAAAAGTGATTCCGTAAGTAGTGG    250

M.kansasii        251 CGAGCGAACGCGGAACATGGCTAAACCGCACGCATGGGTAACCGGGTAGG    300
                      ||||||||||||||||||||||||||||||||||||||||||||||||||
NT47              251 CGAGCGAACGCGGAACATGGCTAAACCGCACGCATGGGTAACCGGGTAGG    300

M.kansasii        301 GGTTGTGTGTGCGGGGTTGTGGGATCGATACGTCTCAGCTCTACCCGGCT    350
                      ||||||||||||||||||||||||||||||||||||||||||||||||||
NT47              301 GGTTGTGTGTGCGGGGTTGTGGGATCGATACGTCTCAGCTCTACCCGGCT    350

M.kansasii        351 GAGGGGCAGTCAGAAAGTGTCGTGGTTAACGGAAGTGGCCTGGGATGGTC    400
                      ||||||||||||||||||||||||||||||||||||||||||||||||||
NT47              351 GAGGGGCAGTCAGAAAGTGTCGTGGTTAACGGAAGTGGCCTGGGATGGTC    400

M.kansasii        401 TGCCGTAGACGGTGAGAGCCCGGTACGTGAAAACCCGGCACCTGCCTTGT    450
                      ||||||||||||||||||||||||||||||||||||||||||||||||||
NT47              401 TGCCGTAGACGGTGAGAGCCCGGTACGTGAAAACCCGGCACCTGCCTTGT    450

M.kansasii        451 ATCAATTCCCGAGTAGCAGCGGGCCCGTGGAATCTGCTGTGAATCTGCCG    500
                      ||||||||||||||||||||||||||||||||||||||||||||||||||
NT47              451 ATCAATTCCCGAGTAGCAGCGGGCCCGTGGAATCTGCTGTGAATCTGCCG    500

M.kansasii        501 GGACCACCCGGTAAGCCTAAATACTCCTCGATGACCGATAGCGGAATAGT    550
                      ||||||||||||||||||||||||||||||||||||||||||||||||||
NT47              501 GGACCACCCGGTAAGCCTAAATACTCCTCGATGACCGATAGCGGAATAGT    550

M.kansasii        551 ACCGTGAGGGAATGGTGAAAAGTACCCCGGGAGGGGAGTGAAAGAGTACC    600
                      ||||||||||||.|||||||||||||||||.||||||||||||.||.||.
NT47              551 ACCGTGAGGGAAAGGTGAAAAGTACCCCGGAAGGGGAGTGAAATAGAACT    600

M.kansasii        601 TGAAACCGTGTGCCTACAATCCGTCAGAGCCCTTTCGTGGGGTGATGGCG    650
                      |||||||||||||.|||||...||||||||||.||..| |||||||||||
```

```
NT47          601 TGAAACCGTGTGCTTACAAGTAGTCAGAGCCCGTTAAT-GGGTGATGGCG   649

M.kansasii    651 TGCCTTTTGAAGAATGAGCCTGCGAGTCAGGGACATGTCGCGAGGTTAAC   700
                  |||||||||.|||||||.||||||||||||||||||||||||||||||||
NT47          650 TGCCTTTTGTAGAATGAACCTGCGAGTCAGGGACATGTCGCGAGGTTAAC   699

M.kansasii    701 CCGTGCGGGGTAGCCGCAGCGAAAGCGAGTCTGAATAGGGCGTATCGCGC   750
                  ||||||||||||||||.|||||||||||||||||||||||||||||||||
NT47          700 CCGTGCGGGGTAGCCGTAGCGAAAGCGAGTCTGAATAGGGCGTATCGCGC   749

M.kansasii    751 GCGAGCGTGTGTAGTGGCGTGTTCTGGACCCGAAGCGGAGTGATCTACCC   800
                  ||||||||||||||||||||||||||||||||||||||||||||||||||
NT47          750 GCGAGCGTGTGTAGTGGCGTGTTCTGGACCCGAAGCGGAGTGATCTACCC   799

M.kansasii    801 ATGGCCAGGGTGAAGCGCGGGTAAGACCGCGTGGAGGCCCGAACCCACTT   850
                  ||||||||||||||||||||||||||||||||||||||||||||||||||
NT47          800 ATGGCCAGGGTGAAGCGCGGGTAAGACCGCGTGGAGGCCCGAACCCACTT   849

M.kansasii    851 AGGTTGAAGACTGAGGGGATGAGCTGTGGGTAGGGGTGAAAGGCCAATCA   900
                  ||||||||||||||||||||||||||||||||||||||||||||||||||
NT47          850 AGGTTGAAGACTGAGGGGATGAGCTGTGGGTAGGGGTGAAAGGCCAATCA   899

M.kansasii    901 AACTCCGTGATAGCTGGTTCTCCCCGAAATGCATTTAGGTGCAGCGTTGC   950
                  ||||||||||||||||||||||||||||||||||||||||||||||||||
NT47          900 AACTCCGTGATAGCTGGTTCTCCCCGAAATGCATTTAGGTGCAGCGTTGC   949

M.kansasii    951 GTGTTTCACCACGGAGGTAGAGCTACTGGATGGCCGATGGGCCCCACTAG  1000
                  ||||||||||||||||||||||||||||||||||||||||||||||||||
NT47          950 GTGTTTCACCACGGAGGTAGAGCTACTGGATGGCCGATGGGCCCCACTAG   999

M.kansasii   1001 GTTACTGACGTCAGCCAAACTCCGAATGCCGTGGTGTATAGCGTGGCAGT  1050
                  ||||||||||||||||||||||||||||||||||||||||||||||||||
NT47         1000 GTTACTGACGTCAGCCAAACTCCGAATGCCGTGGTGTATAGCGTGGCAGT  1049

M.kansasii   1051 GAGACGGCGGGGGATAAGCTCCGTACGTCGAAAGGGAAACAGCCCAGATC  1100
                  ||||||||||||||||||||||||||||||||||||||||||||||||.|
NT47         1050 GAGACGGCGGGGGATAAGCTCCGTACGTCGAAAGGGAAACAGCCCAGACC  1099

M.kansasii   1101 GCCGGCTAAGGCCCCAAAGCGTGTGCTAAGTGGGAAAGGATGTGCAGTCG  1150
                  .||.|||||||.||||||||||||||||||||||||||||||||||||||
NT47         1100 ACCAGCTAAGGTCCCAAAGCGTGTGCTAAGTGGGAAAGGATGTGCAGTCG  1149

M.kansasii   1151 CAGAGACAACCAGGAGGTTGGCTTAGAAGCAGCCACCCTTGAAAGAGTGC  1200
                  |||||||||||||||.|||||||||||||||||||.|.||.|||||||||
NT47         1150 CAGAGACAACCAGGATGTTGGCTTAGAAGCAGCCATCATTTAAAGAGTGC  1199

M.kansasii   1201 GTAATAGCTCACTGGTCAAGTGATTGTGCGCCGATAATGTAGCGGGGCTC  1250
                  |||||||||||||.|||.|||||...||||||||.||||||.||||||||
NT47         1200 GTAATAGCTCACTAGTCGAGTGACACTGCGCCGAAAATGTACCGGGGCTC  1249

M.kansasii   1251 AAGCACACCGCCGAAGCCGCGACAACCGCAAGGTTGGGTAGGGGAGCGTC  1300
                  ||||||||||||||||||||||||||||||||||||||||||||||||||
NT47         1250 AAGCACACCGCCGAAGCCGCGACAACCGCAAGGTTGGGTAGGGGAGCGTC  1299

M.kansasii   1301 CCTCATTCAGCGAAGCTGCCGGGTGACCGGTGGTGGAGGATGGGGGAGTG  1350
                  ||||||||||||||||||||||||||||||||||||||||||||||||||
NT47         1300 CCTCATTCAGCGAAGCTGCCGGGTGACCGGTGGTGGAGGATGGGGGAGTG  1349

M.kansasii   1351 AGAATGCAGGCATGAGTAGCGATAAGGCAAGTGAGAACCTTGCCCGCCGA  1400
                  ||||||||||||||||||||||||||||||||||||||||||||||||||
NT47         1350 AGAATGCAGGCATGAGTAGCGATAAGGCAAGTGAGAACCTTGCCCGCCGA  1399

M.kansasii   1401 AAGACCAAGGGTTCCTGGGCCAGGCCAGTCCGCCCAGGGTGAGTCGGGAC  1450
                  ||||||||||||||||||||||||||||||||||||||||||||||||||
```

```
NT47            1400 AAGACCAAGGGTTCCTGGGCCAGGCCAGTCCGCCCAGGGTGAGTCGGGAC 1449

M.kansasii      1451 CTAAGGCGAGGCCGACAGGCGTAGTCGATGGACAACGGGTTGATATTCCC 1500
                     |||||||||||||||.||||||||.|||||||.|||.||||||||||||.
NT47            1450 CTAAGGCGAGGCCGATAGGCGTAGGCGATGGATAACAGGTTGATATTCCT 1499

M.kansasii      1501 GTACCCGTGTGTGGGCGCCCGTGATGAATCAGCGGTACTAACCACCCAAA 1550
                     ||||||||||||||||||||||||||||||||||||||||||||||||||
NT47            1500 GTACCCGTGTGTGGGCGCCCGTGATGAATCAGCGGTACTAACCACCCAAA 1549

M.kansasii      1551 ACCGGATCGATCACTCCCCTTCGGGGGCGTGGAGGTCTGGGGCTGCGTGG 1600
                     ||||||||||||||||||||||||||||||||||||||||||||||||||
NT47            1550 ACCGGATCGATCACTCCCCTTCGGGGGCGTGGAGGTCTGGGGCTGCGTGG 1599

M.kansasii      1601 AGCCTTCGCTGGTAGTAGTCAAGCGATGGGGTGACGCAGGAAGGCAGCCG 1650
                     ||||||||||||||||||||||||||||||||||||||||||||||||||
NT47            1600 AGCCTTCGCTGGTAGTAGTCAAGCGATGGGGTGACGCAGGAAGGCAGCCG 1649

M.kansasii      1651 TACCAGTCAGTGGTAATACTGGGGCAAGCCAGTAGGGAGAGCGATAGGCA 1700
                     ||||||||||||||||||||||||||||||||||||||||||||||||||
NT47            1650 TACCAGTCAGTGGTAATACTGGGGCAAGCCAGTAGGGAGAGCGATAGGCA 1699

M.kansasii      1701 AATCCGTCGCTCACAAATCCTGAGAGGTGACGCATAGCCGATTGAGGCGA 1750
                     ||||||||||||||||||||||||||||||||||||||||||||||||||
NT47            1700 AATCCGTCGCTCACAAATCCTGAGAGGTGACGCATAGCCGATTGAGGCGA 1749

M.kansasii      1751 ATTCGGTGATCCTCTGCTGCCAAGAAAAGCCTCTAGCGAGCACACACACG 1800
                     ||||||||||||||||||||||||||||||||||||||||||||||||||
NT47            1750 ATTCGGTGATCCTCTGCTGCCAAGAAAAGCCTCTAGCGAGCACACACACG 1799

M.kansasii      1801 GCCCGTACCCCAAACCGACACAGGTGGTCAGGTAGAGAATACCAAGGCGT 1850
                     ||||||||||||||||||||||||||||||||||||||||||||||||||
NT47            1800 GCCCGTACCCCAAACCGACACAGGTGGTCAGGTAGAGAATACCAAGGCGT 1849

M.kansasii      1851 ACGAGATAACTATGGTTAAGGAACTCGGCAAAATGCCCCCGTAACTTCGG 1900
                     |||||||||||||||||||||||||||||||||||.||||||||||||||
NT47            1850 ACGAGATAACTATGGTTAAGGAACTCGGCAAAATGACCCCGTAACTTCGG 1899

M.kansasii      1901 GAGAAGGGGGACCGGAATACCGTGAACACCCTTGCGGTGGGAGCGGGATT 1950
                     ||||||||||||||||||||||||||||||||||||||||||||||||||
NT47            1900 GAGAAGGGGGACCGGAATACCGTGAACACCCTTGCGGTGGGAGCGGGATT 1949

M.kansasii      1951 CGGTCGCAGAAACCAGTGAGAAGCGACTGTTTACTAAAAACACAGGTCCG 2000
                     ||||||||||||||||||||||||||||||||||||||||||||||||||
NT47            1950 CGGTCGCAGAAACCAGTGAGAAGCGACTGTTTACTAAAAACACAGGTCCG 1999

M.kansasii      2001 TGCGAAGTCGCAAGACGATGTATACGGACTGACGCCTGCCCGGTGCTGGA 2050
                     ||||||||||||||||||||||||.||.||||||||||||||||||||||
NT47            2000 TGCGAAGTCGCAAGACGATGTATAGGGGCTGACGCCTGCCCGGTGCTGGA 2049

M.kansasii      2051 AGGTTAAGAGGACCCGTTAACCCGCAAGGGTGAAGCGGAGAATTTAAGCC 2100
                     ||||||||||||||||||||||||||||||||||||||||||||||||||
NT47            2050 AGGTTAAGAGGACCCGTTAACCCGCAAGGGTGAAGCGGAGAATTTAAGCC 2099

M.kansasii      2101 CCAGTAAACGGCGGTGGTAACTATAACCATCCTAAGGTAGCGAAATTCCT 2150
                     ||||||||||||||..|||||||||||..|||||||||||||||||||||
NT47            2100 CCAGTAAACGGCGGCCGTAACTATAACGGTCCTAAGGTAGCGAAATTCCT 2149

M.kansasii      2151 TGTCGGGTAAGTTCCGACCTGCACGAATGGCGTAACGACTTCTCAACTGT 2200
                     |||||||||||||||||||.|||||||.||||||||||||||||||||||
NT47            2150 TGTCGGGTAAGTTCCGACCCGCACGAAAGGCGTAACGACTTCTCAACTGT 2199

M.kansasii      2201 CTCAACCATAGACTCGGCGAAATTGCACTACGAGTAAAGATGCTCGTTAC 2250
                     ||||||||||||||||||||||||||||||||||||||||||||||||||
```

```
NT47             2200 CTCAACCATAGACTCGGCGAAATTGCACTACGAGTAAAGATGCTCGTTAC  2249

M.kansasii       2251 GCGCGGCAGGACGAAAAGACCCCGGGACCTTCACTACAACTTGGTATTGG  2300
                      ||||||||||||||||||||||||||||||||||||||||||||||||||
NT47             2250 GCGCGGCAGGACGAAAAGACCCCGGGACCTTCACTACAACTTGGTATTGG  2299

M.kansasii       2301 TGTTCGGTACGGTTTGTGTAGGATAGGTGGGAGACTGTGAAACCTCAACG  2350
                      |||||||||||||||||||||||.....||||||||||||||||||||||
NT47             2300 TGTTCGGTACGGTTTGTGTAGGANNNNNGGGAGACTGTGAAACCTCAACG  2349

M.kansasii       2351 CCAGTTGGGGTGGAGTCGTTGTTGAAATACCACTCTGATCGTATTGGACA  2400
                      ||||||||||||||||||||||||||||||||||||||||||||||||||
NT47             2350 CCAGTTGGGGTGGAGTCGTTGTTGAAATACCACTCTGATCGTATTGGACA  2399

M.kansasii       2401 CCTAACGTCGAACCCTGAATCGGGTTCACGGACAGTGCCTGGCGGGTAGT  2450
                      ||||||||||||||||||||||||||||||||||||||||||||||||||
NT47             2400 CCTAACGTCGAACCCTGAATCGGGTTCACGGACAGTGCCTGGCGGGTAGT  2449

M.kansasii       2451 TTAACTGGGGCGGTTGCCTCCTAAAATGTAACGGAGGCGCCCAAAGGTTC  2500
                      ||.|||||||||||.||||||||||..|||||||||||||.||||||||.
NT47             2450 TTGACTGGGGCGGTCGCCTCCTAAAGAGTAACGGAGGCGCTCAAAGGTTT  2499

M.kansasii       2501 CCTCAACCTGGACGGCAATCAGGTGGCGAGTGTAAGTGCACAAGGGAGCT  2550
                      ||||||||||||||||||||||||||||||||||||||||||||||||||
NT47             2500 CCTCAACCTGGACGGCAATCAGGTGGCGAGTGTAAGTGCACAAGGGAGCT  2549

M.kansasii       2551 TGACTGCGAGACCTACAAGTCAAGCAGGGACGAAAGTCGGGACTAGTGAT  2600
                      ||||||||||||.||||||||.|||||||.||||||||||||||||||||
NT47             2550 TGACTGCGAGACTTACAAGTCGAGCAGGGTCGAAAGTCGGGACTAGTGAT  2599

M.kansasii       2601 CCGGCACCTCTGAGTGGAAGGGGTGTCGCTCAACGGATAAAAGGTACCCC  2650
                      |||||||||||||||||||||||||||||||||||||||||||.||||||
NT47             2600 CCGGCACCTCTGAGTGGAAGGGGTGTCGCTCAACGGATAAAAGCTACCCC  2649

M.kansasii       2651 GGGGATAACAGGCTGATCTTCCCCAAGAGTCCATATCGACGGGATGGTTT  2700
                      ||||||||||||||.||||.||||||||||.||.|||||||||..|||||
NT47             2650 GGGGATAACAGGCTTATCTCCCCCAAGAGTTCACATCGACGGGGAGGTTT  2699

M.kansasii       2701 GGCACCTCGATGTCGGCTCGTCGCATCCTGGGGCTGGAGCAGGTCCCAAG  2750
                      |||||||||||||||||||.||||||||||||||||.||..|||||||||
NT47             2700 GGCACCTCGATGTCGGCTCATCGCATCCTGGGGCTGTAGTCGGTCCCAAG  2749

M.kansasii       2751 GGTTGGGCTGTTCGCCCATTAAAGCGGCACGCGAGCTGGGTTTAGAACGT  2800
                      |||||||||||||||||||||||||||.||||||||||||||.|||||||
NT47             2750 GGTTGGGCTGTTCGCCCATTAAAGCGGTACGCGAGCTGGGTTCAGAACGT  2799

M.kansasii       2801 CGTGAGACAGTTCGGTCTCTATCCGCCGCGCGCGTCAGAAGCTTGAGGAA  2850
                      |||||||||||||||||.||||||||||||||||||||||||||||||||
NT47             2800 CGTGAGACAGTTCGGTCCCTATCCGCCGCGCGCGTCAGAAGCTTGAGGAA  2849

M.kansasii       2851 ACCTGTCCCTAGTACGAGAGGACCGGGACGGACGAACCTCTAGTGCACCA  2900
                      ||||||||.|||||||||||||||||||.||||..||||||.|||.||||
NT47             2850 ACCTGTCCTTAGTACGAGAGGACCGGGATGGACATACCTCTGGTGTACCA  2899

M.kansasii       2901 GTTGTCCCACCAGGGGCACCGCTGGATAGCTACGTTCGGACAGGATAACC  2950
                      ||||||||||||||||||||||||||||||||||||||||||||||||||
NT47             2900 GTTGTCCCACCAGGGGCACCGCTGGATAGCTACGTTCGGACAGGATAACC  2949

M.kansasii       2951 GCTGAAAGCATCTAAGCGGGAAACCTTCTCCAAGATCAGGCTTCTCACCC  3000
                      ||||||||||||||||||||||||||||||||||||||||||||||||||
NT47             2950 GCTGAAAGCATCTAAGCGGGAAACCTTCTCCAAGATCAGGCTTCTCACCC  2999

M.kansasii       3001 ACTTGGTGGGATAAGGCCCCCCGCAGAACACGGGTTCGATAGGCCAGACC  3050
                      ||||||||||||||||||||||||||||||||||||||||||||||||||
```

```
NT47             3000 ACTTGGTGGGATAAGGCCCCCCGCAGAACACGGGTTCGATAGGCCAGACC   3049

M.kansasii       3051 TGGAAGCTCAGTAATGAGTGAAGGGAACTGGCACTAACCGGCCGAAAACT   3100
                      ||||||||||||||||||||||||||||||||||||||||||||||||||
NT47             3050 TGGAAGCTCAGTAATGAGTGAAGGGAACTGGCACTAACCGGCCGAAAACT   3099

M.kansasii       3101 TACCAACACAAATAATCG   3118
                      ||||||||||||||||||
NT47             3100 TACCAACACAAATAATCG   3117
```
